# Elements of Pathological Anatomy; Illustrated by Colored Engravings and 250 Woodcuts

**Published:** 1846-04

**Authors:** 


### REVIEWS.

---

**Art. III.**—*Elements of Pathological Anatomy;* Illustrated by Colored Engravings and Two Hundred and Fifty Woodcuts. By Samuel D. Gross, M.D., Professor of Surgery in the Medical Institute of Louisville; Late Professor of Pathological Anatomy in the Medical Department of the Cincinnati College; Surgeon to the Louisville Marine Hospital, etc., etc. *Second Edition, thoroughly Revised and greatly Enlarged.* Philadelphia: Ed. Barrington & Geo. D. Haswell. Louisville: James Maxwell, jr. 1845. Super-royal 8vo. pp. 822.

[CONTINUED FROM PAGE 134.]

Resuming our notice of this volume, we come next to Chapter XVII, which treats of *Polypes.* These occur in any part of the mucous system, except the gall-bladder, ureters, and Fallopian tubes. The most common situation is the nose; next to this is the uterus; then comes the maxillary sinus; then the rectum; and, lastly, the vagina. Polypes are likewise found in the inferior animals, especially the horse, ass, cow, and dog; and here they affect the same situations as in the human subject. In regard to their structure, four varieties are observed: the vesicular, gelatinoid or cellular, which is most frequently seen in the nose; the fibrous, most common in the uterus; the vascular, rarer than the two preceding, and found in the rectum, external ear, vagina, and uterus; lastly, the granular, least frequent of any, and found chiefly in the uterus. They are all liable to undergo changes from disease, like other morbid productions; and these changes have been erected into classes by some pathologists. Hence the carcinomatous, lardaceous, fibro-cartilaginous, cartilaginous, osseous, and earthy polypes. The fibrous and granular varieties are liable to malignant degeneration; the others rarely, if ever.

The harder varieties are nearly always solitary; the softer ones frequently multiple, or in clusters. They sometimes co-exist in different parts of the body. Polypes are not new formations, but result from hypertrophy of the mucous membrane alone, or conjoined with some of the textures which it overlies. Thus in different polypes, different tissues predominate: in the fibrous, the fibrous textures of the part; in the vesicular, the mucous membrane and the cellular tissue beneath it; in the granular, the mucous follicles.

Polypes are always covered by mucous membrane, which is liable to various alterations. In the interior, cysts are frequently found, containing serous, purulent or bloody fluids; and even tubercular matter has been seen in nasal polypes. The effects of these tumors on the surrounding parts vary according to their size and situation. In the uterus, even a very small one may give rise to fatal hemorrhages. In the maxillary sinus, they produce immense deformity, by distending that cavity, and encroaching upon the eye, nose, and lachrymal canal. In the larynx, they cause dyspnœa, and alteration or total extinction of voice. Polypes, it need scarcely be said, are endowed with blood-vessels, nerves, and absorbents.

Chapter xviii—Treats of a curious and interesting subject—that of *Hydatids*. Hydatids have the form of a thin vesicle, filled with a clear transparent fluid, and surrounded by a dense fibrous capsule. They infest nearly all classes of animals. In the human subject they are most common in adults and the old, but have been observed in the fœtus. Their existence is brief, occupying at most but a year or two. They have been differently classified by different writers; the author adopts the division of Cloquet, who makes five genera: the *cysticercus*, the *polycephalus*, the *diceras*, the *echinococcus*, and the *acephalocystis*. The first four genera are provided with appendices resembling heads, hooklets, tentacula, or suckers; hence they are sometimes called cephalocysts; they exhibit a very feeble degree of sensibility and motility. The acephalocysts, as the name implies, have no heads, or hooklets, but consist of a simple globular vesicle; they are impassive under stimuli, exhibiting neither motion nor sensibility, their claims to individual life resting upon the power of self-nutrition and of reproduction which they possess in common with the others. Of the cysticercus there are five species: 1. The cellular, found almost exclusively in the hog, in which it constitutes the *measles*, and seen only once or twice in man. 2. The vesicular, observed once in the choroid plexus of an apoplectic subject—common in the ox, sheep, hog, goat, stag, and gazelle. 3. The dicystis, found by Laennec in the lateral ventricles of an apoplectic man. 4. The speckled, observed once by Treutler in the choroid plexus of a young female. 5. The Fischerian, found likewise in the choroid plexus of the human subject, on two occasions, by Fischer, after whom it is named. The polycephalus is rare, and has not yet been found in the human subject; it occurs in the brain, liver, and intermuscular cellular tissue of the inferior animals—when existing in the brain, producing what is called the *staggers*. The diceras is met with in the alimentary canal of the lower animals, and occasionally in that of man. The echinococcus, a genus not admitted by Cuvier, occupies the brain, liver, spleen, and omentum, in the lower animals; in man it has been twice observed in the ventricles of the brain, and once voided in the urine. The most common, as well as the most interesting class, is the acephalocystis. This occurs both in man and the lower animals, and infests particularly the liver, but has been found in every part of the body except the alimentary canal, urinary bladder, and lungs. Cruveilhier divides the acephalocysts into two species, the solitary and the social. The former is most common in the lower animals, and generally exists simultaneously in several organs; the latter is oftenest seen in man, and rarely infests more than a single organ, its favorite seat being the ovary and uterus, from the latter of which immense masses are sometimes expelled. The internal surface of the acephalocyst, and of some other varieties, is often found studded with minute granular bodies, greyish and spherical, which are supposed to be young hydatids; the cyst is then said to be endogenous. Sometimes the granular bodies are found upon the external surface; the entozoon is then said to be exogenous. The former is most frequent in the human subject, the latter in ruminant animals. Occasionally the development takes place between the different tunics of the cyst. A large acephalocyst is sometimes found to contain several smaller ones, situated one within the other, like so many pill-boxes, and progressively diminishing in size. Occasionally, these secondary cysts, by their rapid development, burst the parent cyst. Hydatids are not in immediate contact with the tissues where they are situated, but are surrounded and isolated by a cyst; and often between their parietes and this enclosing capsule, is interposed a soft, pulpy, dirty-looking substance, regarded by Hodgkin as an excrementitious secretion of the hydatid itself.

Whence come these parasites? and what is their organization? These are interesting questions, but in answer to them we have nothing but conjectures more or less plausible. As it respects their origin, the author adopts the opinion of Vitet, that they result from inflammation—an inflammation of a specific nature, "followed by the deposition of a fibro-albuminous substance, or, what is the same thing, a sort of plastic lymph, the particles of which arrange themselves in such a manner as to create an inferior being, an entozooic parasite." The capsule enclosing this parasite, is not derived, as Hodgkin supposes, from the surrounding cellular tissue, inasmuch as it is frequently found where that tissue is almost absent; but is a new formation, like the hydatid itself, developed at the same time with the latter, and differing from it only in the arrangement of its particles. This enclosing cyst is furnished with vessels, in many instances continuous with those of the adjacent parts, in other cases having no such connection, but evidently of new formation. In no case can these vessels be traced into the parietes of the hydatid. Has the latter any vessels? and whence comes the clear transparent fluid it contains when healthy, or the turbid, discolored, and variously altered fluid when diseased? Secretion does not take place in any animal, however minute, without vessels; and hence, if the contents of the hydatid are allowed to be of its own production, it must be provided with vessels. Experiments have been made by Carmichael and Owen, from which it appears that endosmose takes place in these animals; but these experiments only show that the fluids in question *may* gain admittance in that mode, not that they always *do* thus gain admittance. A question still remains: whence come the materials for the nourishment of these beings? This is derived from the vessels of the enclosing cyst, being filtered through the soft pulpy substance which lies between it and the hydatid, and then imbibed by the proper cyst of the latter. Such, are some of the author's conjectures—for they are nothing more, and claim to be nothing more. They are by no means satisfactory to us, nor do they seem altogether consistent with each other.

Klencke, of Berlin, has some novel views concerning hydatids. He considers them as a *living contagious principle*, and has published experiments to show their transmissibility. As they exist in very many animals, not only in the flesh, but likewise in the urine, fæces, milk, menstrual fluid, and even in the blood, there is no scarcity of them in the external world, either as ovules, or individuals more or less completely developed; and once admitted into the human intestinal canal they may find their way thence into different tissues, or the ovules, being excessively minute, may enter the circulation and be transported through the system to any organ of the body that affords a proper *nidus* for their reception. The acephalocysts, according to the same writer, are not distinct from the echinococci, being ova of the latter; and the polycephalous hydatid he views as a sort of polypus, each one of the heads representing a distinct individual being. He has seen the parent vesicle with an appearance of contraction or division, which he regards as commencing fission. The polycephalus is also propagated by buds or gems like the acephalocyst; so that the reproduction of hydatids takes place both by fissiparous and gemmiparous generation.

As the acephalocyst neither feels nor moves, Professor

Owen thinks it doubtful whether they can be referred to the animal kingdom as that kingdom is at present characterized. He goes on to say: "It would then be a question how far its chemical composition forbids us to rank the acephalocyst among vegetables. In this kingdom it would obviously take place next those simple and minute vesicles which in the aggregate constitute the green matter of Priestly (*Protococcus viridis*, *Agardh*); or those equally simple but differently colored *psychodiariæ*, which give rise to the red snow of the arctic regions (*protococcus kermesianus.*) These 'first born of Flora' consist, in fact, of a simple transparent cyst, and propagate their kind by gemmules developed from the external surface of their parent."

The successive development of acephalocysts one within the other, bears a striking similarity to the development of the primitive cells of animal and vegetable structure; for the former like the latter, appear to be nucleated cells from the interior of which new cell-germs are evolved. Now it can be conceived that inflammation might act as a cause of disaggregation to the cells of which the tissues are composed; but it remains to understand how the off-sets thus formed become endowed with individuality, or with a separate and distinct life.

Whether we can trace the immediate causes of hydatids, or not, we know some of the circumstances under which they are produced. Sheep kept in wet clayey pastures, and eating juicy unripe vegetables, are subject to them. The author estimates that one-tenth of the hogs annually slaughtered in the shambles of Cincinnati, are thus affected; and he accounts for it by the fact that these animals "are literally stuffed with fresh corn for five or six weeks before they are sent to market;" hence arises congestion of the portal circle, and inflammation of the liver and other organs, with development of acephalocysts. The cysticerci are developed in rabbits confined in damp coops, shut out from sun and air. And in the human subject, the entozoa generally are most common in moist countries.

Hydatids sometimes disappear without any obvious cause. They may suffer inflammation, leading when acute to suppuration and gangrene; or, when more chronic, to thickening and induration of the parietes, and sometimes to the cartilaginous and bony transformations. To the surrounding parts they prove mischievous by their number and their size; exciting inflammatory action, which goes on to suppuration, softening, gangrene, induration—or to ulceration, by which they make their way through the hardest structures.

Similar to the vesicular worms just discussed, and often confounded with them, but yet different in many important particulars, are the *Serous Cysts* met with in various parts of the body. They form shut sacs, like the natural serous membranes—smooth and in contact with a fluid on the internal surface, rough and adherent on the other; in size, they vary from a mustard-seed to a large melon; and in shape, are more or less globular. Like hydatids they have been observed in nearly all classes of animals; but are most frequent in the old, and, according to the author's experience, more frequent in the female than in the male. The organs which seem to be their favorite seat are the ovaries, liver, kidneys, mammæ, and testicles, to which may be added the brain of old persons. According to their structure, serous cysts may be divided into three kinds: the *simple*, which consists of a thin, delicate, transparent sac, containing a pellucid fluid in all respects similar to the serum of the blood, and provided with long slender vessels evidently derived from the adjacent parts; the *multilocular*, where the cyst is divided into compartments or cells by processes given off from the internal surface and intersecting each other in various ways; the *included*, where instead of cells, the main cyst contains several smaller ones composed of a single lamella, which is evidently a reflection from the original sac. The simple cyst is seen particularly in the ovaries, the fimbriated extremities of the Fallopian tubes, the liver, and the brain—especially in the lateral ventricles of that organ in the old. The multilocular is common in the brain around old apoplectic effusions, in the ovaries, and in the cellular tissue where subjected to pressure, as on the shoulders of porters, and the knees of chambermaids. The included are also found in the ovaries, and in the broad ligaments of the uterus. The contents of these cysts while healthy are clear, transparent, of a sero-albuminous character, and somewhat saline. In a state of disease, this fluid is variously altered—becomes turbid, thickened, discolored—is mixed with blood, flakes of fibrin, and fatty matter; contains substances not found in the healthy body, as pus, tubercle, or substances peculiar to certain parts of it, as cholesterine. In the multilocular cysts several of these substances may be found at the same time in different compartments. Thus, one shall contain serum, another pus, a third blood, a fourth tubercle, or fatty, meliceritous, or atheromatous matter.

These cysts are most frequently the result of a new formation, which is dependent on some perversion of nutrition brought about by inflammatory irritation; or they are constructed out of pre-existing textures—which may be serous, as in enlargement of the Graafian vesicles—or mucous, as in the kidneys and female breast, from obstruction of the excretory ducts—there being then a true transformation. When thus formed the cyst may have several coverings. Thus in the ovaries, the parietes may be separated into three layers, one derived from the serous capsule of the vesicle of De Graaff, another from the proper fibrous coat of the ovary, and a third from the peritoneal coat of that body. Serous cysts are liable to inflammation; the contained fluid undergoes various changes; the capsule becomes dense and fibrous, and irregularly thickened, or is converted wholly or partially into cartilage and bone. The effects of the cyst on the surrounding parts resemble those arising from hydatids.

Chapter xx—Is devoted to the *Heterologous Formations*, and is one of the longest and most elaborate in the book. The term heterologous is used to designate "certain morbid products, of a solid and semi-concrete consistence, which have no resemblance whatever, or at most a very remote one, to the natural, normal, or pre-existing tissues of the body."

The word heteroclite is employed in the same sense. According to the author's views, there are but five of these products, viz: tubercle, scirrhus, encephaloid, colloid, and melanosis. These are considered in as many separate sections. The parasitic animals, as hydatids and worms, and the calcareous concretions, are on the limits of this class of productions, if indeed they do not belong to it. Any arrangement must be imperfect in the present state of the science—

"Indeed, I am not certain that the term heterologous, as applied to these formations, is not altogether ill-chosen, and out of place, since most of them are found, when carefully investigated, to have a very close resemblance, in many of their most essential features, to the normal tissues of the body. Thus, encephaloid bears a striking similitude to the substance of the brain; melanosis, to the coloring matter of the skin; scirrhus, to the dermoid texture; tubercle, at least in some of its forms, to fibro-cartilage; and colloid, to the vitreous humor of the eye."—124, 125.

The common tendency of all these formations is to destroy the tissues in which they are located; hence they have been termed malignant. *Tubercle* is not usually classed among malignant productions; but in view of its extraordinary rapidity, and its fatality—causing one-third of all the deaths that occur—it must be regarded as among the most malignant. The author defines tubercle to be "a small, solid tumor, of an irregularly spherical figure, more or less opake, of a pale yellowish color, seldom exceeding the volume of a pea, susceptible of organization, and composed of a peculiar substance, which, sooner or later, undergoes a process of decomposition." But this definition really includes only two varieties.

Tubercle may occur in almost every organ of the body; but its particular situation varies in a considerable degree according to the age of the subject. From the researches of Lombard, Rilliet and Barthez, Louis, and others, tables of which are given by the author, it may be inferred,

—"first, that, in children, tubercles not unfrequently occur in different parts of the body, without existing in the lungs; secondly, that they are more liable to affect the lymphatic ganglions than in adults; and, thirdly, that they have a tendency to attack a much greater number of organs simultaneously or successively. It is a singular circumstance that the spleen is seldom tuberculized in adults, whilst it is very often affected in children, in the proportion nearly of one to four. From the tables of Louis and Lombard, but particularly from that of the latter, it would appear that the liver is remarkably exempt from this disease at all periods of life; a result which is, however, strikingly at variance with the statement of Rilliet and Barthez, who found this organ affected seventy-one times in three hundred and fourteen cases. Papavoine observed the liver tuberculized in more than one-fourth of his autopsies, or in fourteen children out of fifty. The intestines are affected with nearly equal frequency at both periods of life."—127, 128.

The period most liable to this deposit is between twenty and forty. It may occur, however, at any age, and even during fœtal life. The law promulgated by Louis in 1825, has been verified by every subsequent observer, viz: that after the fifteenth year, tubercles are never found in any organ unless they exist at the same time in the lungs. Another well established law, is, that in children tubercles are secreted more rapidly and in greater abundance than in adults. Rilliet and Barthez find that the periods of childhood most liable to them are from six to ten years and a half, then from eleven to fifteen, next from two to five, and lastly from one to two and a half. Barlow thinks "that any organ is most liable to become the seat of tuberculous deposit, when its vascular and functional activity bears the greatest ratio to the other organs of the body." He supports this view by reference to the greater frequency of tubercle in the brains of children, or when that organ is relatively the largest; also in the chylopoietic viscera of older children; and in the lungs of youths at the time when these organs are attaining their complete development. It is further supported, he thinks, by the suspension of tubercular disease during pregnancy, and the absence of tubercles from such portions of the lung as have been compressed in cases of pneumo-thorax following phthisis.

This deposit is observed in many of the lower animals: in these, as in man, it occurs at all ages, and its most frequent site is the lung. It is also observed in birds, reptiles, and insects.

The composition of tubercular matter has been examined by numerous observers, and with very different results. The preference is given by the author to the analysis of Hecht, who found twenty-six parts of albumen, twenty-two of gelatine, thirty-four of fibrin, and thirty of water, in one hundred and twelve parts of dense tubercular matter. Thenard found in one hundred parts of crude tubercle ninety-eight of albumen, the remainder consisting of salts of lime and soda, with a trace of iron. Lombard found nearly the same proportions. But in chalky tubercle the proportions were just reversed, the salts being ninety-six and the animal matter four.

"The discrepancies in the results of the above analyses may be accounted for by supposing that the chemical constitution of tubercular matter varies, as no doubt it does, not only in the different stages of its existence, but also in different individuals, in different situations, and in different parts even of the same organ. It is reasonable to conclude, also, that it is modified, more or less, by the state of the solids and fluids, or, in other words, by the cachexy, or constitutional peculiarity leading to its formation. In all these respects tubercular matter bears the closest resemblance to serum, lymph, and pus, which are often remarkably altered in their chemical and physical properties by the nature of the affected tissue, the state of the system, and the concomitant inflammation. In the inferior animals, the composition of this substance presents, perhaps, still greater variety than in the human subject. In the ox, there is always an unusual predominance of earthy salts, and hence the extraordinary brittleness which characterizes the morbid product. In the turtle I have seen the tubercular substance of the color and consistence of calcareous moss, or what, in mineralogical language, is termed tufa. In the sheep, horse, and some other quadrupeds, on the contrary, the animal matter is generally much greater than the saline, especially in the early stage of the disease.'—129.

There is also a difference of opinion as to what particular texture tubercle is seated in. According to Carswell it affects more particularly the mucous membranes; Cruveilhier thinks it is originally deposited in the ultimate venous radicles, as a result of capillary phlebitis; others, in the elastic cellular tissue. It may occur on the surface of a membrane, or in the parenchyma of the different organs.

Tubercle is seen in different forms. Most frequently it occurs in little grains like millet seed, whence the name *miliary;* these by coalescing form tumors varying in size from a pin-head to a billiard-ball. These little masses are sometimes enveloped by a distinct capsule, and then constitute the *encysted* tubercle; this variety is very rare. Again, instead of being collected into masses of greater or less size, the heteroclite matter is *infiltrated* into the substance of an organ, and varies from the consistence of jelly (*gelatiniform infiltration* of Laennec) to that of fibro-cartilage. Lastly, instead of being diffused in the texture of a part, it is poured out on some surface, as that of a mucous membrane, in layers of greater or less thickness, constituting the *stratiform* variety, which stands next to the miliary in frequency. A variety of miliary tubercle which has attracted a good deal of attention, are the *grey granulations* of Bayle. These occur isolated or in groups, frequently in immense numbers; they adhere to the surrounding tissue and are encircled by red vessels which occasionally extend into them. After a time they lose their greyish opaline tint, and assume the appearance of the common miliary tubercle, with which they are nearly always associated—the change beginning in the centre. Bayle thought they were of a cartilaginous nature; Laennec regarded them as nascent tubercles, an opinion in which Louis and Rokitansky concur. Chapman, with apparent gravity, thinks to decide the question by calling them *tuberculoid.*

"To me the grey granulation seems to be merely a variety of the common tubercle, modified by the action of the affected part, the state of the blood, and the condition of the general health. Serum, lymph, and pus are modified in this manner, and why should not tubercle be? To maintain that the grey granulation is merely a nascent tubercle, is, to say the least, not very philosophical. That it is liable to be transformed into the common yellow tubercle is certain, but that death may, and often does take place, before any change is effected, is equally true. The grey granulation may precede the yellow tubercle, or it may be deposited simultaneously with it. Under whatever circumstances it is found, it always contains a disproportionate quantity of animal matter, and is endowed with much greater power of resisting the influence of such agents as have a tendency to destroy it. It is evidently the product of a more healthy action; it indicates a better state of the solids and fluids; in a word, it is a more plastic, organizable substance than common tubercle."—133.

As to the origin of tubercle, the author looks upon it as altered blood-plasma, the effusion of which takes place under the influence of inflammatory irritation, preceded in nearly all cases by the tubercular dyscracia or cachexy.

"In the former edition of this work I expressed the opinion that tubercles are always of inflammatory origin, and a more thorough examination of the subject since has only tended to confirm this conclusion. The concomitant action is usually very languid, or so mild and imperceptible that extensive mischief is often done before the patient is aware of it. In this respect, the development of tubercles resembles that of a chronic abscess, which is rarely characterized by any of the ordinary phenomena of phlegmasia.

"The doctrine of the inflammatory origin of this disease is countenanced, if not actually established, by the following circumstances:

"First, by chemical analysis. The experiments of Hecht, the most satisfactory yet made, afford nearly equal parts of albumen, gelatine, and fibrin; substances which, whether they occur alone, or in combination with each other, are always to be regarded, when found upon the surfaces or in the interstices of the organs, as the result of inflammatory irritation.

"Secondly, tubercular matter, as before stated, bears a very great resemblance to spoiled, degraded, or cacoplastic lymph, which is an acknowledged product of inflammation.

"Thirdly, the deposit is often excited by cold, especially when conjoined with moisture, and by unwholesome, indigestible, or innutritious food. Dyspepsia frequently leads to the same result. By the former are produced internal congestions; by the latter, a poor and impoverished state of the the blood, so favorable to the development of tubercle.

"Fourthly, in many cases the disease is attended or preceded by hyperæmia, or active congestion. This often happens in the lungs, the pleura, peritonæum, and lymphatic ganglions.

Fifthly, the doctrine of the inflammatory origin of this deposition derives great plausibility from what occurs in the inferior animals, from mechanical irritation. In the experiments of Cruveilhier, Kay, and Saunders, well-characterized tubercles were produced in a very short time, simply by dropping mercury into the trachea. Similar effects are frequently witnessed in miners, needle-grinders, and weavers, who habitually inhale gritty or irritating matter. Persons of this description are peculiarly prone to phthisis.

"Sixthly, scirrhus and encephaloid, in fact, all carcinomatous growths, are of inflammatory origin. All these products are derived from the blood, they gradually become softened, and they ultimately destroy the tissues in which they are situated. Scirrhus, like tubercle, contains fibrin, gelatine, and albumen.

"Seventhly, there is no appreciable deposit, secretion, or effusion in any of the shut sacs, cells, or cavities of the body, which is not, strictly speaking, the result of inflammatory action, though this may be so slight as not to attract attention, or to be unattended by the ordinary phenomena of that process."—135, 136.

The author also contends for the organization of tubercles. Lugol has detected blood-vessels in this deposit; Kingston has seen them with the microscope; and Macartney has demonstrated them by injection. The following circumstance we ourselves witnessed:

"In a lung which Dr. Bayless, at my request, last summer injected with size colored with vermilion, a number of tubercles exhibited the clearest possible evidence of vascularity. On cutting into a mass of this kind, a tubercle, about the size of a duck-shot, and of a light greyish color, was divided, from the centre of which the artificial fluid, which was still warm, ran in a small jet or stream, precisely as blood flows from a divided vessel."—136.

Additional evidence is furnished by the fact that these bodies often become yellowish in jaundice; also by their softening, which generally begins in the centre, and by their conversion into cretaceous or earthy masses, a change which may begin at any part. This organization, however, is not constant. The peculiar modified character of tubercle in some situations, as upon the mucous membranes of the bronchial tubes, seminal vesicles, and uterus, is opposed to it. In these situations the deposit is more curdy and friable, and contains more earthy ingredients.

"Within the last ten years I have examined not less than ten or a dozen specimens of organized tubercles of the kidney, spleen, peritonæum, and lungs, mostly of young subjects. The tubercles were of the miliary kind, and numerous vessels, loaded with florid blood, could be seen shooting into them in every direction, many of them penetrating a considerable distance into their substance. Their vascular supply would thus seem to be derived from the tissues in which they are deposited; and this, in the generality of cases, is no doubt true; nevertheless, there is reason to believe that they occasionally possess a self-organizing power, analogous to that of the adventitious membranes of the splanchnic cavities."—137.

The vessels forming the proper circulation of tubercle have never been seen by the author; but he asserts positively that he has frequently traced vessels into the mass from the surrounding tissues. Nor have they been injected.

"Granting this to be true, and what, it may be asked, does it prove? Are we reduced to the necessity of denying the vitality of a structure, because we cannot succeed in throwing foreign substances into its vessels? If this be a fair criterion, then we must conclude that the cornea, the crystalline lens, the membrane of Jacob, the inner coat of the arteries and veins, the endocardium, and the arachnoid tunic of the brain, together with the chorion and amnion, are not organized. But who would be so silly as to do this, when daily observation demonstrates that these textures are abundantly supplied with vessels, nerves, and absorbents, though none of these tissues can be detected by inspection, no matter what methods we resort to for the purpose."—138.

Microscopically, as well as chemically, tubercle seems to differ according to the part where it is deposited, and the period at which it is examined. When recent it exhibits cells and granules, like those of plastic lymph, but no fibres are perceptible. These cells possess but a low power of development, and instead of tending to a higher organization they more frequently recede to the granular or amorphous condition. Thus, at a later period, and after the mass has increased, it is found to consist almost wholly of granular matter, with some irregular granular corpuscles, and shrivelled and imperfect cells. This is especially seen in the caseous tubercle. This increase of the mass is rarely due to intra-cellular development, or the successive evolution of cell-germs, but to the super-addition or juxta-position of new particles. Now, between coagulable lymph, and chalky or friable tubercular matter, there occurs every variety; and as the departure from the former is less, the plastic force will be greater; so that we can conceive how sometimes tubercle may grow by intussusception, and how sometimes it may be self-organized as contended for by the author.

Softening of tubercle, according to the author's views, is analogous to slow suppuration. The deposit by and by produces irritation in the tissues where it is situated; this irritation is soon communicated to the heteroclite mass, which, in consequence of its weak vital endowments, readily yields. Of this softening, and the excavations consequent upon it, and of some other changes in tubercles, we shall speak in connection with another chapter.

*Melanosis* has been seen by the author very seldom in his numerous autopsies, and therefore he thinks it very rare in this country. Like tubercle it occurs at all ages, though it is most frequent in the old; like it, it affects the lower animals; like it, also, it is hereditary both in the human subject and the lower animals. It is more common in white horses and cattle than colored ones. Barruel and Hecht found it to contain fibrin, united with the coloring matter of the blood, and three distinct fatty substances. Foy has found in it albumen, fibrin, a carbonized principle supposed to be altered cruor, water, iron, and salts of lime, soda, and magnesia. The microscope shows it to consist of a fibrous network in the meshes of which are pigment cells, elongated and sometimes caudate, containing yellowish and blackish granules. It is collected in masses (tuberoid form); spread out in sheets (lamellated) and streaks (ramiform); deposited in points (punctiform); diffusely infiltrated.

Several melanotic masses may be agglomerated, constituting a rough and lobulated tumor, which in man rarely exceeds the size of the closed hand, but in the horse attains the weight of many pounds. These tumors have a thin covering formed out of the surrounding textures. They are also intersected by fibrous processes sent off from this covering or derived from the cellular tissue in which the morbid mass was originally deposited. Veins and arteries, the former predominating, ramify over the surface of the tumor, and are distributed to its interior upon the fibrous intersections; the veins are large and tortuous, and both arteries and veins are so weak that they give way in attempts to inject them. The melanotic matter itself contains no vessels, or, in other words, is not organized. On serous surfaces these tumors are sometimes pedunculated like polypes, and are always surrounded by a cyst. Rarely the melanotic matter is found in a *fluid* state, presenting the color and consistence of black ink; it has been thus observed in the serous cavities, lungs, and urinary organs.

"The *tissues* most prone to the melanotic deposition are, beyond all comparison, the cellular and adipous. Large masses of this substance are often met with under the skin, particularly of the trunk, in the mediastinal cavities, in the folds of the mesentery and omentum, and around the kidneys. In horses, the subcutaneous substance of the buttock, anus, vulva, and tail, is a very common seat of the disease. Of the different organs, the liver, lungs, spleen, eye, kidneys, and ovaries, may be enumerated as being most frequently affected. The lymphatic ganglions, especially those of the bronchiæ, are also very liable to it. The bones, fibrous and serous membranes, the pancreas, arteries, and salivary glands, are seldom implicated; while the brain and spinal cord, the nerves, the veins, the muscles and their tendons, the cartilages, the synovial and mucous membranes, the uterus and mammary glands, the thyroid and thymus bodies, the prostate, testicle, seminal vesicle, and supra-renal capsule, enjoy almost an entire immunity from its invasion. Melanosis of the heart has been noticed by Gohier, Fawdington, Breschet, Halliday, Cruveilhier, Cullen, and Carswell; but this is the only portion of the system of involuntary muscles in which this disease has hitherto been found."—145.

As we have a tuberculous, so we may have a *melanotic*, diathesis. Cases are recorded where, either simultaneously or successively, a great number of organs and tissues presented this lesion.

"One of the most remarkable of the kind was published some years ago by Dr. Norris, an English physician. In this instance, not only was the external surface extensively studded with black tumors, but immense numbers were seen scattered over the stomach, intestines, mesentery, and omentum; the lymphatic ganglions, kidneys, pancreas, and liver were also more or less affected; the lungs were thickly mottled throughout the greater part of their texture; and the heart was literally encrusted with them, both externally and internally. The brain was healthy, but the dura mater was deeply involved."—146.

Melanosis is frequently combined with scirrhus, rarely with tubercle. When alone, it may remain stationary for a long time, giving rise to no inconvenience save what is caused by its size and situation. This has led many to believe that *per se* it is not malignant. When the melanotic tumor ulcerates an intractable sore is produced, having hard ragged edges, and discharging black, inky matter, mixed with more or less of blood, pus, or thin fluid from the adjacent structures. When removed, the tumors re-appear in the same place, or in some internal organ. The disease does not seem to be communicable by contact, or inoculation.

"Of the *causes* of this disease, and of the states of the system which predispose to it, nothing is known with any degree of certainty. That the melanotic matter is derived immediately from the blood, both anatomical examination and chemical analysis abundantly show; but how far, or in what respect, this fluid is altered before the deposition is effected, are points in the history of this disease concerning which pathology and physiology are equally silent and undetermined. If we remember that melanosis is essentially composed of the same elements as the coloring matter of the skin and of the choroid coat of the eye, we may be allowed to suppose that this substance, existing in an unnatural quantity in the blood, is deposited, by an aberration of the nutritive functions of the vessels, into organs and tissues in which it is not found in the normal state. Noack regards the black matter as a secretion from the veins, an opinion in which he is joined by Dr. Hodgkin. This view, however, is entirely inadmissible on physiological grounds, inasmuch as there is no instance, so far as we know, except in the liver, in which the veins perform such an office."—146, 147.

We come next to *Scirrhus.* A good deal of confusion exists among pathologists as to the nature of this formation; partly because all malignant diseases whatever have been termed cancer; and partly because scirrhus, besides presenting some varieties, often co-exists with encephaloid. To avoid this confusion, the author defines scirrhus to be "a hard, crisp, opake substance, of a light greyish color, with dull yellowish, fibrous intersections, organized, liable to lancinating pain, occurring for the most part after the middle period of life, and passing sooner or later into ulceration."

Like the morbid products we have already noticed, scirrhus presents itself in different forms; as a tumor solitary or otherwise, an infiltration, or a lamella. The infiltrated form is infrequent, and occurs most often in the lungs, liver, uterus, kidney, and bones; the lamellated affects the submucous cellular tissue of the œsophagus, stomach, and bowels.

"In the *tuberoid* form, the most common, as we have just stated, of all, the heterologous substance forms small circumscribed nodules, the number of which, as in the liver, is sometimes very great, and the consistence of which varies between fresh pork and fibro-cartilage. Their size and shape are much influenced by the nature of the tissues in which they are developed, and by the resistance which is offered to their progress. Single tumors of this kind are rounded, ovoidal, or conical; when, on the contrary, several are agglomerated together, they are generally very irregular, angular, and more or less lobulated. In their size they vary between a mustard-seed and an adult head, their average being that of a billiard-ball, a lemon, or an orange."

"A scirrhous tumor creaks under the knife, is opake, firm, inelastic, and of a white bluish color, with various shades of grey, rose, and drab. These tints are most conspicuous when there is an admixture of bile, blood, or pus, as sometimes happens when the heterologous matter is very old. Thin slices of it are found to be semi-transparent, flexible, and elastic; when dried, they exhibit nearly all the properties of the horny tissue. Fibrous intersections, generally of a slight yellowish color, are seen to pervade the diseased mass, starting from the centre as their common nucleus, and radiating thence towards the circumference. These lines are merely the remains, in most cases, of the cellular substance of the affected part, and are often so arranged as to resemble very closely the fibrous structure of an unripe pear or turnip. A creamy-looking fluid is occasionally incorporated with the heteroclite mass, and constitutes the most decided evidence of its carcinomatous nature."—148, 149.

These growths occasionally contain hydatids. Serous cysts are oftener found, their contents consisting of thin, gummy, meliceric matter, and even pus. Effusions of blood are also observed, and these are thought by some to precede the formation of the cysts. A characteristic of this growth, is the absence of an enclosing capsule; the cellular membrane around old tumors is sometimes condensed, but never forms a proper covering. Scirrhus is subject to some variations in structure, and these have received different names. The *mammary sarcoma*—so called from its resemblance to the female breast—is lobulated, and intersected by fibrous bands of a dull white, bluish grey, or pale straw color; the *pancreatic*—named from its resemblance to the pancreas—consists of lobules, resolvable into granules, and separated by bands of cellular tissue; the *lardaceous*—resembling a section of fresh pork—has little of the fibrous or linear arrangement of ordinary scirrhus, and the malignant disposition is not so marked in it, as it often remains in the crude state for years; the *reticular*, one of the most common, distinguishable by its white or bluish reticular figures, large bulk, and tendency to become lobulated. The solid chemical constituents of this matter, according to Foy, are albumen (42 parts in 100), white and red fatty matter, fibrin, iron, and salts of lime, soda, magnesia, and potash. The solid materials found by Hecht in matter from a scirrhus breast were gelatine, fibrin, fatty matter, and a small quantity of albumen; in matter from a scirrhous uterus, they were gelatine, fibrin, and fatty matter. These differences can only be reconciled by supposing that the composition of this matter, like that of tubercle to which it is analogous, varies according to the part of the body where it is deposited, and the stage of the disease.

Scirrhus rarely appears before the age of *thirty*, and this marks it from all the other heterologous formations. Its favorite period is between the fortieth and fiftieth years. It is hereditary. Very often it succeeds to some external violence, to syphilitic disease, suppression of the menses, and repulsion of herpetic eruptions. Quite as often there is no assignable cause.

"Scirrhus sometimes attacks a considerable number of organs in the same individual, either simultaneously, or in gradual succession. In females, it is not uncommon to find both the breast and the uterus involved at the same time. It not unfrequently co-exists with encephaloid, tubercle, melanosis, and hydatids.

"The parts of the body most liable to scirrhus are such as have a glandular structure. In females, it is most common in the breast and uterus; in males, in the testicle, penis, stomach, and rectum. In both sexes the lips, nose, and liver may be mentioned as frequent seats of it. The spleen, lungs, and kidneys are seldom affected. It is also extremely rare in the bones; and it is doubtful whether it ever occurs in the cartilages, in the serous, synovial, and fibrous textures, and in the muscles of voluntary life."—151.

According to Elliotson it attacks primarily parts not essential to life, and glands the functions of which have ceased, or have never been performed. It affects particularly the breasts of women past child-bearing, and particularly the breasts of those who have never had any children. It affects the breasts, uterus, ovaries, and testes; parts, he remarks, "not necessary to life, being all for the sake of the next generation, and not for ourselves,—except, indeed, as a gratification!"

In relation to the proximate cause, Dr. Gross does not agree with Adams, that scirrhus depends on the presence of a parasitic animal, the *carcinomatous* hydatid; nor with Hodgkin, that it arises from the growth and multiplication of serous cysts. He believes it to arise, like tubercle, from a specific inflammation which produces a plastic effusion analogous to the fibrin of the blood.

"The effusion thus produced takes place in the cellular element of our organs, the proper structure of which it gradually transforms, effaces, or destroys. That this is the case, is sufficiently evinced by what happens in the liver, kidney, and pancreas. Cases occasionally occur in which the heterologous substance can be discovered in different stages of its development, so as to enable us to determine the manner in which it is deposited. Thus, in the liver, the scirrhous matter generally appears in very minute, circumscribed points, corresponding with the granulations which are so abundantly found here in the natural state. At first, there is merely a change of color, the granulations exhibiting a pale greyish aspect, without the slightest deformity or augmentation of volume. At a somewhat later period, the little tumors are observed to be of a white milky hue, hard, dense, crisp, opake, irregularly spherical, and perfectly devoid of the original structure. Now these alterations, it is quite plain, can only be accounted for on the assumption, that, in proportion as the heterologous matter is deposited into the cellular texture of the acini of the liver, their proper parenchymatous substance, whatever it may be, together with their vessels, is obliterated by absorption, the pressure which the accidental secretion produces being fully adequate to bring about this result. Similar phenomena are to be witnessed, as scirrhus is being developed in other organs."—155.

Scirrhus is generally slow, especially when it occurs in the aged. A scirrhous breast at forty-five will run a much more rapid course than one at sixty or seventy. The irritation which it excites in the surrounding structures is also of the chronic character, leading to induration and contraction, and hence forming hard, knotty, corrugated swellings. Scirrhus, like tubercle, undergoes softening, which may commence in any part of the morbid mass; but ulceration usually sets in before it is completed. The emaciation, as remarked by Williams, accompanying the latter stages, is more extreme than in any other disease. The tissues and organs become exsanguine, and suffer both in composition and bulk. We conclude our notice of this section with the following description of an external scirrhous growth:

"Scirrhus is recognized by its circumscribed character, its hardness and incompressibility, and by the peculiar nature of the accompanying pain. The hardness is greater than that of any normal texture, save the cartilaginous and osseous. The pain is usually of a gnawing, lancinating kind, darting through the swelling in different directions, and coming on in irregular paroxysms, which, as the disease progresses, increase both in frequency and violence, as well as in duration. Sometimes it is prurient, hot, burning, or scalding, and is then commonly more permanent. At first, the tumor is movable, distinctly circumscribed, and the skin over it perfectly natural; by and by, however, it contracts adhesions, and soon becomes fixed and less defined, at the same time that its cutaneous covering experiences various alterations, both as respects its smoothness, its color, and its consistence."—156.

*Encephaloid*—the medullary sarcoma, fungus hæmatodes, soft cancer, medullary fungus, and spongoid inflammation of various authors—is allied to the last affection, with which, and also with tubercle, melanosis, hydatids, and other morbid growths, it often coexists. Unlike scirrhus, however, it occurs in early life as well as late, and is remarkably rapid in its progress. The lamellated and infiltrated forms are infrequent.

"In the tuberoid *variety* the heterologous matter appears in the form of a circumscribed tumor, from the size of a pea to that of an adult head. In its shape it is generally irregularly rounded, ovoidal, or even quite flat, according to the amount of pressure exerted upon it by the surrounding parts. It is composed of different lobules, which are enveloped by a thin covering, and separated from each other by delicate membranous partitions. The outer covering, evidently derived from the neighboring cellular tissue, is usually not more than half a line in thickness, easily torn, semi-transparent, and of a light rose color. From its inner surface are detached numerous processes, which, dipping into the morbid growth in various directions, form so many cavities for the reception of the new deposit. These septa, which are sometimes remarkably rough and shreddy, always become more obvious after the pulpy mass is squeezed out. The cells which they form by their intersections are subject to much variety, and hence the peculiar lobulated shape which characterizes the morbid growth when occurring in parts that offer little or no obstacle to its extension."—156, 157.

The external covering is sometimes of new formation. The morbid growth is liberally endowed with blood-vessels, which are extremely large and tortuous, the veins being most numerous. Cavities containing pus, serum, or sanious fluid, are often found in the interior; and sometimes clots of blood, from rupture of the vessels, which, like those of melanosis, are exceedingly fragile. The color is generally cineritious, but runs through various shades of red and white. The consistence varies from that of cream to that of fibro-cartilage; but generally is about equal to that of the fœtal brain. Both color and consistence often vary not only in different growths, but in different parts of the same growth. Names have been given to some of the variations of structure: thus we have the hæmatoid (fungus hæmatodes of Hey) composed partly of erectile tissue, and resembling the placenta, or a clot of blood; the solanoid or potato-like; the nephroid or kidney-like; the napiform or turnip-like; and the hyaline, or, as the author calls it, the fasciculated. In chemical composition encephaloid does not differ from scirrhus, except in the presence of osmazome, and the smaller quantity of salts.

This disease occurs at all periods from fœtal life to old age; but belongs especially to early life. Of one hundred cases, collected by the author, much the largest proportion was below the fortieth year. Its most favorite seats are the eyes, bones, testicles, liver, lymphatic ganglions, and the subcutaneous cellular tissue. It has also been seen in the veins of the liver, kidney, and uterus, and may be engrafted on other growths. Encephaloid of the eye is almost peculiar to childhood. A remarkable feature of the disease is its rapid reproduction. When a tumor has been removed, the disease re-appears in the same spot, in the adjacent structures, or in the internal organs. Travers states he never knew a patient, upon whom he had operated for this affection, survive four years. It is also apt to follow the removal of a scirrhous tumor. As to its origin nothing is known. The author conjectures it to depend upon an aberration of secretion.

"The circumstance that this substance has been found in the blood, is of no small moment in the discussion of this question. It would lead to the inference that, when the sanguine fluid is surcharged with it, the cerebral matter, instead of being deposited in the brain, spinal cord, and nerves, is poured out in the meshes of the cellular element of one or more of the organs of the body. This conjecture is so much the more plausible, as encephaloid disease often arises without any assignable cause, is sometimes decidedly hereditary in its tendency, and almost always exists in several situations at the same time. Chemical analysis, also, comes to our aid here, bringing to light the important fact that the cerebral tissue and the heterologous deposit are essentially alike in their character."—162.

Analogy, he thinks, also supports this view. For example, cholesterine, peculiar to the biliary organs, has been found in a hydrocele, a tumor under the tongue, an abscess of the jaw, an ovarian cyst, and in other places.

"Encephaloid disease, after having attained a certain development, may remain stationary for years, unaccompanied by the slightest uneasiness, until the part receives some injury, when it often grows with frightful rapidity. When seated in the subcutaneous cellular tissue, the tumor that is thus formed is at first quite movable, smooth on the surface, and devoid of sensation; but gradually, as the enlargement progresses, it becomes stationary, irregularly lobulated, elastic to the touch, and more or less painful. If allowed to proceed, the diseased mass has a tendency to open and protrude, generally by ulceration, sometimes by sloughing, and occasionally by the bursting of an abscess situated in its interior. In either case, the exposed surface presents a dark reddish fungous appearance, is highly sensitive, extremely vascular, very prone to hemorrhage, and constantly bathed with a thin, fœtid, irritating, sanious fluid, the quantity of which is sometimes quite profuse. In many instances pure blood is effused, caused by a rupture of some of the vessels of the morbid growth; and this may be so obstinate and copious as gradually to destroy the patient. Occasionally there is a discharge of thin, glairy fluid, resembling the white of egg. Such sores, besides being always highly disagreeable, never heal, from the inability of the parts to form healthy granulations. Sometimes the ulcerated mass sloughs as completely away as if it were dissected out; but these cases are uncommon, and are soon followed by a re-production of the heterologous substance."—164.

Meanwhile the lymphatic glands of the neighborhood become affected, either by sympathy or direct absorption of matter; whilst the vital powers speedily give way under all the symptoms of the cancerous cachexy.

The last of the heterologous formations is the *Colloid.* Carswell speaks of it under the head of hard cancer; Hodgkin thinks it identical with encephaloid. The author believes its characters sufficiently distinctive for separate consideration. He thus points out these characters:

"Colloid is composed of two distinct elements, bearing to each other the relation of containing and contained parts, and differing, consequently, very widely in their physical and chemical properties, as well as in their origin and mode of arrangement. The first may be considered as the fundamental structure, the base, or frame-work, inasmuch as it gives form and solidity to the whole mass. It is made up of a fibrous tissue, hard, firm, slightly elastic, and of a dull whitish, or pale greyish color, the band-like filaments of which intersect each other in every direction, so as to enclose areolæ, cells, or spaces, calculated to contain the jelly-like matter that is deposited within them. The vacuities thus formed present every intermediate size, between a grain of sand and a common marble. In a specimen in my possession the smallest cells are hardly as large as the most delicate pin-head; some are as big as peas, and a few are of the volume of a Lima bean. In their figure they are rounded, ovoidal, or angular; many of the most capacious are multilocular, or divided by their fibrinous septa into different compartments. In regard to their number, the loculi are too variable to admit of any definite statement. Hundreds frequently exist upon a surface not more than an inch square, and, in masses of large size, the probability is that there are myriads, many of them so minute as to be invisible with the naked eye."—166.

These cells are lined by a delicate membrane of a serous nature, connected to the fibrous structure by short cellular tissue; they communicate with each other.

"The other grand constituent of this morbid growth is an unorganizable product, the consistence of which varies from that of soft jelly, or a solution of starch, isinglass, or arrow-root, to that of custard, half-dissolved glue, or semi-concrete albumen. It is generally of a pale straw color verging on green, perfectly clear, transparent, slightly tremulous, and somewhat tenacious or clammy, In the older cells it is sometimes as firm as moist cheese or the white of a boiled egg, opake, and of a light yellowish hue, interspersed with dark points. It has also been found of a reddish color, similar to that of currant jelly, and Cruveilhier has described a variety of it which is of a pearly whiteness, with a granular fracture and feel, and the chemical constitution of caseum. Whatever, however, may be its appearance or consistence, it has seldom any adhesion to the containing cyst, and is therefore always easily enucleated. It is only when the matter is very hard, or intermixed, as it sometimes is, with flakes of lymph, that it is detached or scraped away with difficulty. Müller with the aid of the microscope twice observed acicular crystals in the jelly-like matter of preparations which had been preserved in spirits of wine; and on another occasion he saw caudate corpuscles in a colloid cancer of the mammary gland." —167.

Colloid is plainly organized, but whether its vessels are of new formation, is not known. No analysis has been made of it. The jelly-like matter contains no gelatine or casein, and undergoes no change in spirits of wine.

Colloid occurs in masses, disseminated through an organ, or solitary; and as an infiltration. The tumors are occasionally of large size; the author has one weighing about twenty-five pounds. This growth is limited in its seat; the structures most liable to it are the alimentary canal, particularly the stomach and rectum, the omentum, ovary, lower jaw, and bones of the extremities. It occurs most frequently from the thirty-fifth to the fiftieth year. It has been seen by the author in quadrupeds.

"How this disease originates, or what the causes are under the influence of which it is developed, we are entirely ignorant. Its progress is usually slow, except when the tumor is very vascular, when it is apt to grow with great rapidity. It manifests no disposition to ulcerate, as is the case with scirrhus and encephaloid; is never the seat of hemorrhage or much pain; the general health usually holds out well; and the countenance rarely acquires that sallow, cadaverous hue so common in some of the other forms of carcinoma."—170.

The microscopic character of the last three growths is essentially the same. They all consist of nucleated or nuciferous cells, contained in a fibrous or areolar structure. In scirrhus the fibrous matter is very abundant, forming the bands or striæ visible to the naked eye. In encephaloid, the fibrous structure is more delicate, and the cells, which are very numerous, are globular, like those of scirrhus, or elliptical, or spindle-shaped and caudate. In colloid, the fibrous structure is also apparent, but the cells are not juxta-posed as in scirrhus; they are encased one within the other, whilst free nuclei or cyto-blasts are observed in the areolæ. The cellular structure, as has been seen, prevails in melanosis. And there can scarcely be a doubt, that if tubercle were examined at a sufficiently early period, it would constantly be found to possess a cellular organization. Even in the caseous tubercle, after changes have unquestionably taken place, imperfect cells and corpuscles are observed. Indeed, if we must adopt the "cell-theory," will it not be found applicable to all growths, abnormal as well as normal? Such a result may be confidently anticipated from further researches.

We here close our analysis of the first part of Professor Gross's volume. We have occupied more space than it was our intention to; but it so grew upon us, so many things presented themselves for notice, that we felt, not that we ought to say less, but that we ought to say much more. In a subsequent paper or two, we shall notice the second part devoted to special pathology. C.

